# Risk of bias reporting in the recent animal focal cerebral ischaemia literature

**DOI:** 10.1042/CS20160722

**Published:** 2017-10-13

**Authors:** Zsanett Bahor, Jing Liao, Malcolm R. Macleod, Alexandra Bannach-Brown, Sarah K. McCann, Kimberley E. Wever, James Thomas, Thomas Ottavi, David W. Howells, Andrew Rice, Sophia Ananiadou, Emily Sena

**Affiliations:** 1 Centre for Clinical Brain Sciences, University of Edinburgh, Chancellor’s Building, Little France Crescent, Edinburgh, U.K.; 2 SYRCLE, Nijmegen Institute for Health Sciences, Radboud University Medical Center, Nijmegen, Netherlands; 3 Evidence for Policy and Practice Information and Coordinating (EPPI)-Centre, Social Science Research Unit, UCL Institute of Education, University of London, London, U.K.; 4 School of Medicine, University of Tasmania, Hobart, TAS, Australia; 5 Pain Research, Department of Surgery and Cancer, Imperial College, London, U.K.; 6 School of Computer Science and National Centre for Text Mining, University of Manchester, 131 Princess Street, Manchester, U.K.

**Keywords:** animal models, lacunar, middle cerebral artery occlusion, research improvement, risks of bias, text mining

## Abstract

Background: Findings from *in vivo* research may be less reliable where studies do not report measures to reduce risks of bias. The experimental stroke community has been at the forefront of implementing changes to improve reporting, but it is not known whether these efforts are associated with continuous improvements. Our aims here were firstly to validate an automated tool to assess risks of bias in published works, and secondly to assess the reporting of measures taken to reduce the risk of bias within recent literature for two experimental models of stroke.

Methods: We developed and used text analytic approaches to automatically ascertain reporting of measures to reduce risk of bias from full-text articles describing animal experiments inducing middle cerebral artery occlusion (MCAO) or modelling lacunar stroke.

Results: Compared with previous assessments, there were improvements in the reporting of measures taken to reduce risks of bias in the MCAO literature but not in the lacunar stroke literature. Accuracy of automated annotation of risk of bias in the MCAO literature was 86% (randomization), 94% (blinding) and 100% (sample size calculation); and in the lacunar stroke literature accuracy was 67% (randomization), 91% (blinding) and 96% (sample size calculation).

Discussion: There remains substantial opportunity for improvement in the reporting of animal research modelling stroke, particularly in the lacunar stroke literature. Further, automated tools perform sufficiently well to identify whether studies report blinded assessment of outcome, but improvements are required in the tools to ascertain whether randomization and a sample size calculation were reported.

## Introduction

There has been much recent interest in strategies to improve the usefulness of findings from biomedical research [1]. This has been occasioned by a growing realization that many ‘findings’ in the published literature cannot be replicated, either in single replication attempts [2,3] or in formal replication studies ([4], https://osf.io/e81xl/wiki/home/).

There are a number of potential reasons for this, including low positive predictive values of the originator study, publication bias, heterogeneity of treatment effects due to the variable presence of unknown modifying variables, overreliance on tests of statistical rather than biological significance and flexibility in study protocols and statistical analysis plans. Notwithstanding these possibilities, it may be that findings reported from laboratory research are overstated because individual studies are at risk of bias, leading to an overstatement of treatment effects. Across the animal modelling of a range of neurological conditions it is apparent that studies which do not report, for instance, randomization of animals to group or the blinded assessment of outcome, give inflated estimates of treatment effect [5]. Identifying the true prevalence of measures to reduce the risk of bias is not straightforward because it may be that scientists have taken account of these measures but not, for whatever reason, reported this in their manuscript. Recognition of this has led to the development of reporting standards for *in vivo* research including the ARRIVE guidelines [6] and the Landis checklist [7].

**Figure 1:**
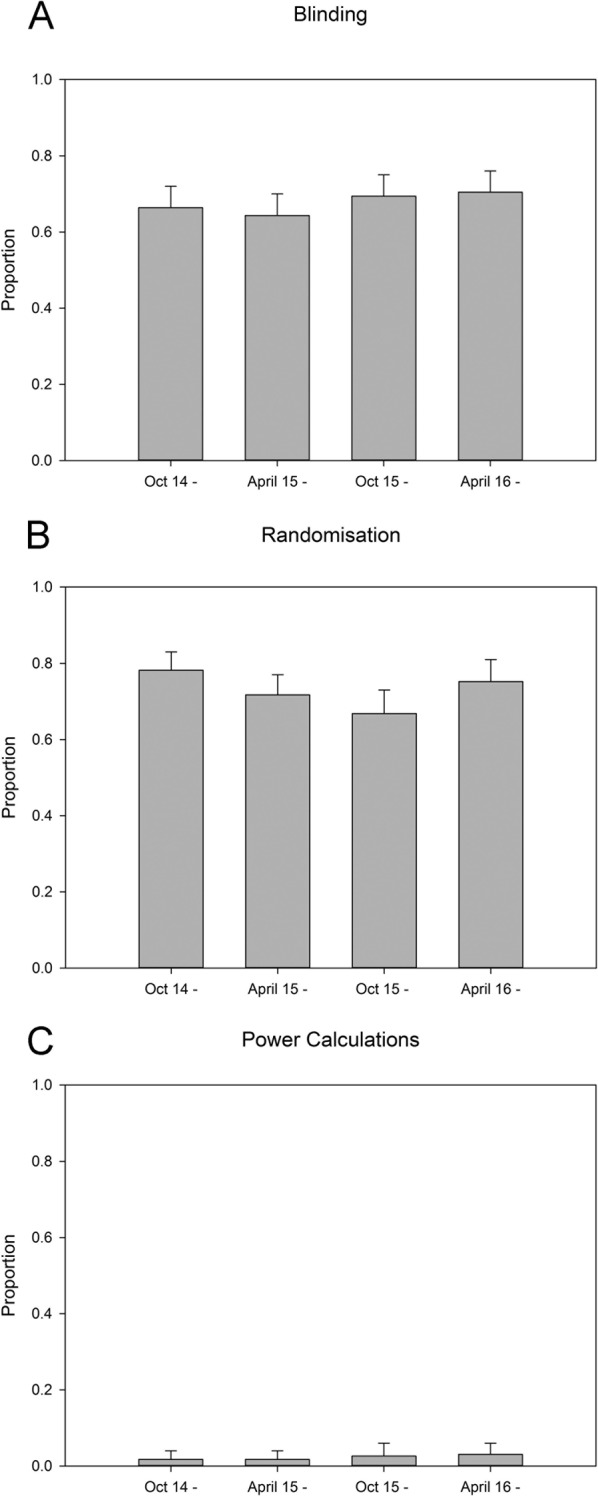
Reporting of risks of bias in the recent MCAO literature. Reporting of (**A**) blinding, (**B**) randomization and (**C**) sample size calculation in the middle cerebral artery occlusion literature in 6-month epochs to October 2016. Values represent mean and 95% Clopper–Pearson exact confidence intervals.

**Figure 2:**
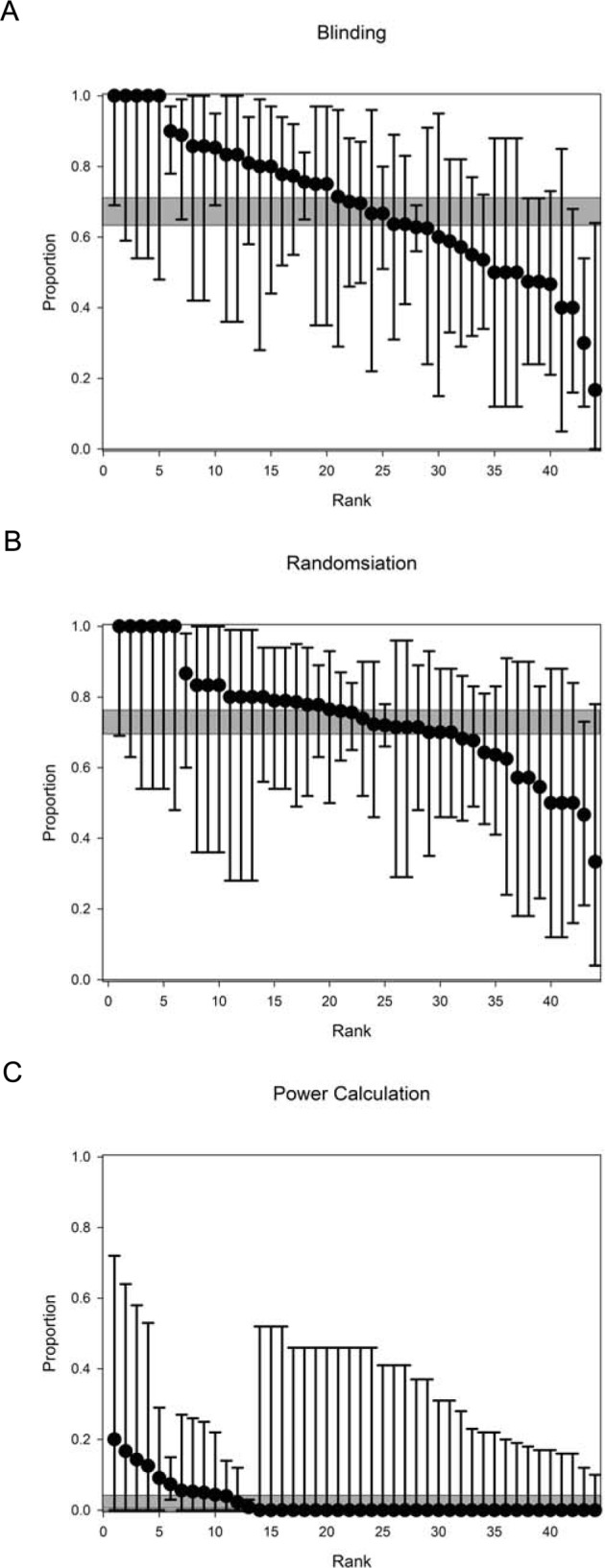
Reporting of risks of bias by Journal. Reporting of (**A**) blinding, (**B**) randomization and (**C**) sample size calculation in the middle cerebral artery occlusion literature in the 2 years to October 2016 by journal of publication for journals with five or more relevant manuscripts, ranked by performance. Values represent mean and 95% Clopper–Pearson exact confidence intervals. The grey shaded bar represents the 95% confidence interval of the overall performance across 918 manuscripts.

**Figure 3:**
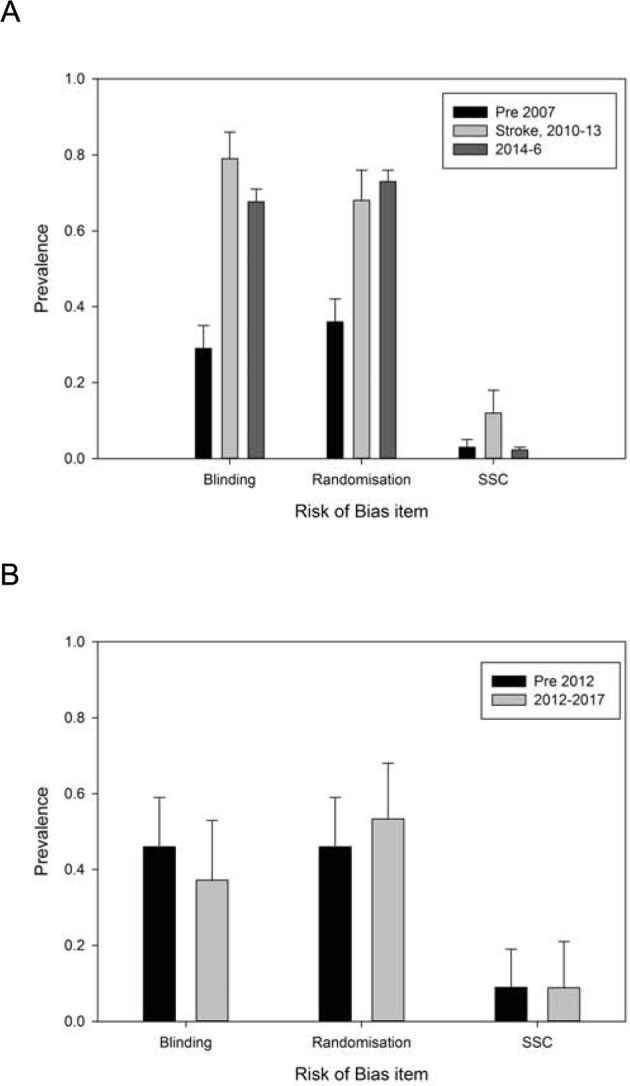
Reporting of risks of bias in the recent literature compared with previous estimates. (**A**) Reporting of blinding, randomization and sample size calculation in the MCAO literature collated from systematic reviews and summarized in 2007 in Sena et al. [8] (column 1), from the journal Stroke from 2010 to 2013 reported by Minnerup et al. [10] (column 2), and from 2014 to 2016 reported here (column 3). (**B**) Reporting of blinding, randomization and sample size calculation in the experimental lacunar stroke literature from the systematic review of 2012 [12] (column 1), and from 2012 to 2017 reported here (column 2).

The *in vivo* stroke research community have been amongst the first to adopt best practice guidelines for the conduct and reporting of animal studies. Following the demonstration that the reporting of risks of bias in *in vivo* stroke research was low [8], Good Laboratory Practice guidelines for *in vivo* stroke research were published in 2009 [9] and were adopted as editorial policy by a number of Journals including ‘Stroke’. Subsequently, Minnerup et al. [10] have shown a substantial increase in the prevalence of reported randomization of group allocation, blinded conduct of the experiment, blinded assessment of outcome and sample size calculations in the years from 2010 to 2013. Recent research suggests that improvement may been limited to specific journals reporting predominantly stroke research [11]. In 2014, Pedder et al. [12] reported a systematic review of animal studies in lacunar stroke published between 1992 and 2011, where the reporting of measures to reduce risks of bias was in general better than had been observed in the middle cerebral artery occlusion (MCAO) literature in 2007 [8]. It is not known, however whether the quality of reporting in lacunar stroke has improved since that time Figures 1–3.

Ascertainment of improvement in reporting might serve as an important outcome measure for efforts to improve performance at the level of a journal, funder, or institution, as part of a research improvement activity. However, one problem with this approach is that ascertainment of reporting of measures to reduce risks of bias in published work is a time-consuming process, requiring review of full-text by at least two independent assessors to minimize risk of human error and taking around 1 h for each manuscript. We have therefore set out to develop text mining tools to automatically ascertain the reporting of key measures to reduce risks of bias in published works describing animal research modelling of MCAO and lacunar stroke.

## Methods

This development work was not guided by a study protocol. Criteria for successful performance of a text mining system were developed at a consensus meeting organized by the SLIM (Systematic Living Information Machine) consortium in April 2016 (prior to the commencement of this project), which recommended that a tool performing with sensitivity and specificity of at least 0.80 could make a valuable contribution.

### Identification of relevant manuscripts

For MCAO studies, we searched PUBMED on 4 October 2016 with the terms <middle cerebral artery occlusion> OR <MCAO>, limited to animals, with a date of record completion later than 4 October 2014. For lacunar stroke studies, we searched PUBMED on 11 January 2017 using the terms employed in Pedder et al., limited to a deposition date between 6 March 2012 (the date of the search which informed the Pedder review) and 11 January 2017 for the terms ((< Biological Models> OR <Animal Disease Models> OR <Animal Models> OR <Animals> OR <mammals> OR <primates> OR <mice> OR <rats>) AND (<cerebrovascular disorders> OR <basal ganglia cerebrovascular disease> OR <brain ischemia> OR <cerebrovascular accident> OR <brain infarction> OR <brain hypoxia ischemia> OR <brain edema> OR <cerebrovascular accident> OR <vascular dementia> OR <intracranial arterial diseases> OR <intracranial vasospasm>) AND (<Stroke* [TW]> OR <cerebrovasc* [TW]> OR <cerebral vasc* [TW]> OR <cerebral* [TW]>) AND (<micro vessel [tw]> OR <small vessel [tw]> OR <perforat* vessel [tw]> OR <arteriole [tw]> OR <lacunar stroke [tw]> OR <lacunar infarct [tw]> OR <small stroke* [tw]> OR <small occlusion* [tw]> OR <small disease* [tw]> OR <micro stroke* [tw]> OR <micro occlusion* [tw]> OR <micro disease* [tw]> OR <lacun* infarct* [tw]> OR <lacun* lesion* [tw]> OR <lacun* stroke* [tw]> OR <small infarct* [tw]> OR <small lesion* [tw]> OR <small stroke* [tw]> OR <subcortical infarct* [tw]> OR <subcortical lesion* [tw]> OR <subcortical stroke* [tw]> OR <deep infarct* [tw]> OR <deep lesion* [tw]> OR <deep stroke* [tw]> OR <silent infarct* [tw]> OR <silent lesion* [tw]> OR <silent stroke* [tw])) NOT (<heart [tiab]> OR <bone [tiab]> OR <eye [tiab]> OR <lung [tiab]> OR <kidney [tiab]> OR <liver [tiab]> OR <renal [tiab]> OR <intestine* [tiab]> OR <spinal [tiab]> OR <pulmonary [tiab]> OR <hepatic [tiab]> OR <global [tiab]> OR <AD [tw]> OR <PD [tw]> OR <Alzheimer* [tw]> OR <Parkinson* [tw]> OR <epilepsy [tw]> OR <MS [tw]> OR <Multiple Sclerosis [tw]), limited to animals. Each of these search results was screened by a single investigator using the NC3Rs/CAMARADES SyRF facility (app.syrf.org.uk) for inclusion. We also extracted the journal in which the work was published. Full-text pdf articles were retrieved through the reference management software EndNote X8 using our institutional full-text subscriptions, and subsequently converted to text files using PDF Extractor SDK.

### Development of ‘regular expressions’

In the context of a systematic review of studies describing animal models of psychosis, we collected phrases associated with the reporting of randomization, blinded assessment of outcome and sample size calculations and used these to develop target regular expressions (“RegEx”s). These were informally tested through application to a second dataset of 1173 full-text publications describing *in vivo* research where risks of bias had been previously extracted [13], and no changes were made. We then applied these tools to the converted full-text of stroke publications using the Grepl function in R, resulting in a call of ‘true’ or ‘false’ for each of the measures to reduce risk of bias. We then conducted manual (‘gold standard’) ascertainment of reporting of measures to reduce risk of bias for each publication. To compare the text analytic with human ‘gold standard’, we calculated sensitivity (effectiveness of the tool in identifying positives), specificity (effectiveness of the tool in identifying negatives) and accuracy (number of publications correctly labelled as a proportion of the total number of publications).The R code is available at GitHub (link provided on publication).

We present the prevalence of reporting of randomization of animals to experimental groups, blinded assessment of outcome and sample size calculation, along with exact binomial confidence intervals (derived using cii in STATA) for each dataset and, for the MCAO literature, in quartiles of PUBMED accession date and by journal. Least squares linear regression was used to understand the correlation between time of publication and the reporting of measures taken to reduce the risk of bias.

## Results

In 2259 studies reporting animal models of psychosis, the RegEx had performed above our pre-defined performance criteria (sensitivity of 0.80 and specificity of 0.91 for randomization, sensitivity of 0.83 and specificity of 0.95 for blinding, and sensitivity of 1.00 and specificity of 0.96 for sample size calculation). In a second dataset of *in vivo* studies from leading U.K. institutions [13], the tool was somewhat less efficient in calling papers as randomized (sensitivity of 1.00 and specificity of 0.62), but blinding and sample size calculation ascertainment were still above our successful performance criteria level of 0.80 (sensitivity of 0.88 and specificity of 0.98 for blinding, and sensitivity of 0.88 and specificity of 0.97 for sample size calculation).

For the MCAO search we identified 1311 publications, of which 1152 met our inclusion criteria. We were able to retrieve full-text pdfs for 918 of these.

The RegEx called 670 of 918 MCAO studies (73%) as reporting randomization, 621 (68%) as reporting the blinded assessment of outcome and 21 (2%) as reporting a sample size calculation. Compared with manual assessment, sensitivity was 1.00 (randomization), 0.99 (blinding) and 0.26 (sample size calculation), with specificity of 0.67 (randomization), 0.77 (blinded assessment of outcome) and 0.99 (sample size calculation). Accuracy was 87% (randomization), 90% (blinded assessment of outcome) and 95% (sample size calculation) (Table 1).

**Table 1:** Summary of performance of RegEx compared with gold standard of manual human ascertainment

	Prevalence (RegEx)	Prevalence (gold standard)	Sensitivity	Specificity	Accuracy
**MCAO** (*n*=918)					
Randomization	73%	60%	1.00	0.67	87%
Blinding	68%	59%	0.99	0.77	90%
Sample size calculation	2%	6%	0.26	0.99	95%
**Lacunar** (*n*=46)					
Randomization	67%	37%	1.00	0.48	67%
Blinding	61%	53%	1.00	0.81	91%
Sample size calculation	4%	9%	0.50	1.00	96%

Accuracy is calculated as the number of publications correctly labelled as a proportion of the total number of publications.

Reporting of randomization and of sample size calculations was highest in the last quartile and lowest in the first quartile, but using least squares linear regression we found no significant change over time. In journals represented in the dataset by five publications or more, reporting of randomization ranged from 33% to 100% (median 76%), of blinded outcome assessment from 17% to 100% (median 70%), and of sample size calculations from 0% to 17% (median 0%). Overall, reporting of these measures to reduce risk of bias showed substantial improvements from those observed in a selection of stroke systematic reviews reported in 2007. In the 50 publications carried in the journal Stroke, the performance in 2014–2016 was comparable with that reported from 2013 by Minnerup.

For the lacunar search we identified 492 publications, of which 61 met our inclusion criteria and of which we were able to retrieve full-text for 46. This lower rate of inclusion (12% vs 87% for MCAO) reflects the reduced sensitivity of the search terms used, reflecting in turn the heterogeneity in the terms used to describe animal experiments modelling lacunar stroke.

The RegEx called 31 of 46 lacunar studies (67%) as reporting randomization, 28 (61%) as reporting the blinded assessment of outcome and 2 (4%) as reporting a sample size calculation. Compared with manual ascertainment, sensitivity was 1.00 for each of randomization and blinded assessment of outcome, and 0.50 for sample size calculation, with specificity of 0.48 (randomization), 0.81 (blinded assessment of outcome) and 1.00 (sample size calculation). Accuracy was 67% (randomization), 91% (blinded assessment of outcome) and 96% (sample size calculation). True prevalence of randomization was 37%, of blinding 53% and of sample size calculations 9%. These are essentially unchanged from the Pedder review of the lacunar stroke literature up to 2012, showing no improvements in reporting of measures to reduce risk of bias over time. No journals were represented in the dataset by five publications or more.

## Discussion

There have been improvements in reporting of measures to reduce risks of bias in the MCAO literature over the last 10 years, but we did not observe similar improvements in the *in vivo* lacunar stroke literature. For both MCAO and lacunar stroke the reporting of a sample size calculation remained low, and there remains substantial room for improvement. Within the MCAO literature performance differed between journals, for instance, in journals contributing five or more publications the rate of reporting of randomization ranged from 33% to 100%, of blinding from 17% to 100%, and of sample size calculation from 0% to 17%. There were no substantial differences, in aggregate, in 239 manuscripts published in journals publishing fewer than five included publications compared with 679 manuscripts published in journals publishing five or more included publications (blinding 63% (less than 5) vs 69% (5 or more), randomization 72% vs 73%, sample size calculation 1% vs 3%).

While it has been argued that an experiment not guided by a sample size calculation is not in itself at risk of bias, we consider the presentation of a sample size calculation to give much greater confidence in the results presented. Firstly, in the presence of publication bias, underpowered experiments will lead to a literature that overstates observed effects [14]. Secondly, a sample size calculation gives greater confidence that the experiment is presented as originally planned, rather than group size being incrementally increased until an arbitrary level of statistical significance is achieved. Finally, the positive predictive value of an experiment is increased when a study has adequate statistical power [15], and without a sample size calculation this is less likely to be the case.

Our study has limitations. Firstly, screening and gold standard risk of bias annotations were performed by a single investigator, and this may have led to errors. Secondly, we were only able to perform data extraction for manuscripts where full-text pdf was available for automated retrieval or pdf could be converted to text, and it may be that the reporting of risks of bias is different in journals for which we do not enjoy an institutional subscription. Moreover, the automated tool did not take into account the existence of any supplementary materials that might have been associated with a publication and may have contained the reporting of methodology pertaining to the measures to reduce risk of bias. Such supplementary materials are not always returned during automatic text retrieval; but in any case reporting guidelines usually recommend that such items be covered in the main text.

Finally, the performance of the RegEx for randomization and for sample size calculations did not perform as well for stroke studies as had been the case in other datasets. Problems related mainly to ‘overcalling’ of blinding and randomization (low specificity) and ‘undercalling’ of sample size calculations (low sensitivity). It may be that there are domain specific differences in language used, and these will be incorporated into further iterations of the RegEx used.

Future work will include the integration with text mining tools which support screening (such as app.syrf.org.uk) and reduce the amount of annotations required by using topic analysis and active learning [16,17]; and crowdsourced annotations performed in the context of reviews using the SyRF platform will contribute to the pool of annotated data available for tool development.

However, for the reporting of the blinded assessment of outcome our RegEx performed at or around the level which had previously been determined as the threshold above which it could be used for the automated ascertainment of the reporting of measures to reduce risk of bias in *in vivo* research. Such ascertainment, providing as it does almost real-time reporting of the performance of a journal (or an institution, a funder or a nation), might serve as an indicator of changes in the quality of published research, and be used to guide audit for improvement activity, for instance the application of Plan-Do-Study-Act cycles [18] to research activity.
